# Visco-Hyperelastic Characterization of the Equine Immature Zona Pellucida

**DOI:** 10.3390/ma14051223

**Published:** 2021-03-05

**Authors:** Elisa Ficarella, Mohammad Minooei, Lorenzo Santoro, Elisabetta Toma, Bartolomeo Trentadue, Marco De Spirito, Massimiliano Papi, Catalin I. Pruncu, Luciano Lamberti

**Affiliations:** 1 Dipartimento di Meccanica, Matematica e Management, Politecnico di Bari, 70125 Bari, Italy; elisa.ficarella@poliba.it (E.F.); s.m.minooei@poliba.it (M.M.); lorenzo.santoro@poliba.it (L.S.); elisabetta.toma@poliba.it (E.T.); bartolomeo.trentadue@poliba.it (B.T.); 2 Istituto di Fisica, Università Cattolica del S. Cuore, 00168 Roma, Italy; marco.despirito@unicatt.it (M.D.S.); massimiliano.papi@unicatt.it (M.P.); 3 Department of Mechanical Engineering, Imperial College London, Exhibition Rd., London SW7 2AZ, UK; 4 Department of Design, Manufacturing & Engineering Management, University of Strathclyde, Glasgow G1 1XJ, UK

**Keywords:** equine zona pellucida, nanoscale mechanical characterization, atomic force microscopy, visco-hyperelasticity, inverse analysis

## Abstract

This article presents a very detailed study on the mechanical characterization of a highly nonlinear material, the immature equine zona pellucida (ZP) membrane. The ZP is modeled as a visco-hyperelastic soft matter. The Arruda–Boyce constitutive equation and the two-term Prony series are identified as the most suitable models for describing the hyperelastic and viscous components, respectively, of the ZP’s mechanical response. Material properties are identified via inverse analysis based on nonlinear optimization which fits nanoindentation curves recorded at different rates. The suitability of the proposed approach is fully demonstrated by the very good agreement between AFM data and numerically reconstructed force–indentation curves. A critical comparison of mechanical behavior of two immature ZP membranes (i.e., equine and porcine ZPs) is also carried out considering the information on the structure of these materials available from electron microscopy investigations documented in the literature.

## 1. Introduction

Atomic force microscopy (AFM) is a well-established approach to the mechanical characterization of cells and biopolymer networks [1,2,3,4,5,6,7,8,9,10]. Calibration of nanoindentation measurements [11], sensitivity of finite indentation response to probe geometry [12,13], cell shape [14,15] and residual stresses [16,17] have also been studied. The typical assumption made in AFM investigations on biological materials is to have elastic behavior, which may range from linear elasticity to hyperelasticity.

Viscoelastic effects are negligible if quasi-static AFM tests are performed [18]. However, viscous forces may drive the nanoindentation response of biopolymer networks even at low indentation rates [19]. Experimental determination and modeling of viscoelastic behavior of soft biological materials is well documented in the literature (see, for example, refs. [5,10,13,20,21,22,23,24,25,26,27,28,29,30,31,32,33]). Cellular membranes comprising polymeric networks are typical examples of viscoelastic materials. In this regard, Lamberti et al. [13,29,30,31] carried out several studies on the mechanical response of zona pellucida (ZP) membranes of bovine, porcine and equine species. The ZP is the extracellular coat surrounding mammalian oocyte and it plays a crucial role during oogenesis, fertilization and preimplantation development [34,35,36]. Knowing the mechanical response of the ZP allows us to identify key elements in sperm–ZP interactions, which are very useful in optimizing artificial fertilization and assisted reproduction protocols. It is hence of paramount importance to accurately assess the viscoelastic behavior of the ZP.

However, several issues must be addressed when the goal is to characterize the viscoelastic behavior of a very complex structure like the ZP, especially if the investigation is carried out at the nanoscale and one considers the inherently high complexity of cell nanomechanics. First, which nonlinear elasticity model should be selected for this soft biological matter? Second, which viscous model should be selected? Last, which is the best way to combine the elastic and viscous components into a model that can accurately reproduce the mechanical behavior of the ZP? It should be noted that these questions are very basic and recur for any time-dependent material or structure regardless of whether on is dealing with medicine or biology applications.

A common feature to mammalian ZPs is their polymeric chain network structure, which suggests using a hyperelastic constitutive law inherently developed for describing the mechanical response of polymeric chains. Lamberti et al. [4,13,16,29,30,31] analyzed several hyperelastic constitutive models and found the eight-chain Arruda–Boyce model [37] to be the best hyperelastic model for describing the mechanical response of immature, mature and fertilized ZP membranes under finite indentation. This was a direct consequence of the fact that the AB model inherently simulates the mechanical response of polymeric chains.

As far as it concerns the choice of a suitable viscous model and its integration into the hyperelastic model, it should be noted that there is no exhaustive study on how to discriminate material nonlinearities for the ZP. Lamberti et al. [13,29,30,31] successfully utilized a classical approach combining the Arruda–Boyce hyperelastic model with a Prony series expansion of the dimensionless relaxation modulus. By comparing finite element results with the AFM data gathered at different indentation rates, it was possible to separate hyperelastic and viscous components. Material properties extracted from inverse analysis were independent of indentation rate; the largest standard deviation on properties (i.e., 10% found for the shear modulus) was fully consistent with the statistical dispersion of experimental data. Furthermore, a very good balance between reliability and computational cost of finite element simulations was reached. Interestingly, in the case of immature porcine ZP, it was shown that viscous effects become more significant for sharp indenters [13]. Furthermore, the limit indentation rate below which it is possible to neglect viscous effects was found to change linearly with the radius of curvature of the AFM probe. 

The goal of this study is to characterize the visco-hyperelastic behavior of a very soft material, the immature equine ZP, and compare the identified behavior to that of a “similar” material, the immature porcine ZP. The classical AFM processing analysis used in [19] indicated that the immature equine ZP is about 10 times weaker than the immature porcine ZP. The values of apparent Young’s modulus derived for the two ZPs from AFM measurements were found to be always in the ratio of 10:1 regardless of the selected value of indentation rate that ranged from 0.5 to 10 μm/s. This “similarity” in the ratio of stiffness values over the whole range of indentation rates occurred because the mathematical model used for expressing the relationship between indentation force and indentation depth depends only on the apparent Young’s modulus. However, in [31], the apparent Young’s modulus derived from the Arruda–Boyce hyperelastic model (i.e., using a more accurate description of material nonlinear behavior) was found to be almost the same for the two materials under the assumption that viscous behavior could be described by a single-term Prony series. Furthermore, unlike porcine ZP, it was not possible to define a clear relationship between limit indentation rate and probe geometry for the equine ZP. This indicates that equine and porcine ZPs actually exhibit a substantially different mechanical response.

In order to address the issues mentioned above, in the present research, the amount of nonlinearity entailed by the viscous part of the hyperelastic model is increased in the attempt to identify the mechanical properties of the immature equine ZP in a more accurate way than [31]. Furthermore, different hyperelastic models are also evaluated to verify the suitability of the Arruda–Boyce model for the new time-dependency scheme hypothesized here. The identified mechanical behavior for the equine ZP is also critically compared with the one of porcine ZP in view of information on the specific structures of these two materials available from electron microscopy studies reported in the literature. Finally, the sensitivity of ZP’s mechanical response to probe geometry is analyzed in order to find the range of the limit indentation rate. 

It should be noted that the investigation phases outlined above for the equine ZP are absolutely general and hence apply to the mechanical characterization of any soft material comprising a polymeric structure and exhibiting a highly nonlinear response in the viscoelastic range. Hence, the contribution of this study goes well beyond the simple analysis/characterization of the biological system represented by the ZP cellular membrane, which is modeled as a visco-hyperelastic material.

Another important contribution of the article will be to show how “similar” mechanical behaviors inferred from simplified viscoelastic models become substantially different when time-dependency is properly modeled. Although this aspect may seem trivial, it must be noted that the inherent complexity of visco-hyperelasticity often forces analysts to make simplifying assumptions.

The paper is structured as follows. Section 2.1 summarizes the most important data given in [19] that are used as a starting point for the present study. Section 2.2 and Section 2.3, respectively, describe the finite element model simulating the AFM experiments and the inverse analysis procedure based on nonlinear optimization used for extracting the visco-hyperelastic properties of the ZP. Sensitivity analysis is outlined in Section 2.4. Section 3 presents and discusses the results obtained in this study. Finally, Section 4 summarizes the main findings of this study and outlines directions of future research.

## 2. Materials and Methods

### 2.1. AFM Measurements and Preliminary Evaluation of Mechanical Properties of Equine ZP

The experimental data taken as targets in the identification process were extracted from [19]. That reference documents the nanoindentation experiments originally conducted by two authors of the present study. Immature equine ZP specimens were submitted to nanoindentation tests at different indentation rates of 0.5, 1, 2, 4, 6, 8 and 10 μm/s, respectively. In the experiments, indentation depth was selected so as to have a maximum reaction force of 1.02 nN for all specimens and it decreased as indentation rate increased: from 1020 nm at 0.5 μm/s to 416 nm at 10 μm/s. Atomic force microscopy (NanowizardII, JPK, Berlin, Germany) was combined with an optical microscope (Axio Observer, Zeiss, Jena, Germany). The AFM probe was an ultrasharp silicon nitride cantilever with a tip radius of 10 nm (CSC16, MikroMash, San Jose, CA, USA).

The classical Hertz model was utilized in [19] to determine the value of apparent Young’s modulus *E*_AP_, assuming that the AFM tip is a rigid cone and the ZP a semi-infinite, isotropic and homogeneous elastic body. The indentation force *F*(*δ*) was related to the indentation depth *δ* by the classical expression:

(1) $$F\left(\delta \right)=\frac{2E_{\mathrm{AP}}\tan\left(\gamma \right)}{\;\left(1-\nu^{2}\right)}\delta^{2}$$

where Poisson’s ratio *ν* was set equal to 0.33 while the half opening angle of tip apex *γ* = 20° was accurately measured by electron microscopy. The apparent elastic modulus of the material was found to increase linearly with the indentation rate, from about 3 kPa for 0.5 μm/s to about 15 kPa for 10 μm/s. However, as mentioned in the Introduction section, such a large variation in elastic properties does not appear to be inherent to the immature equine ZP and was hence critically interpreted in this study in view of the visco-hyperelastic behavior of this soft material.

### 2.2. Finite Element Simulation of the Nanoindentation Process

**Figure 1:**
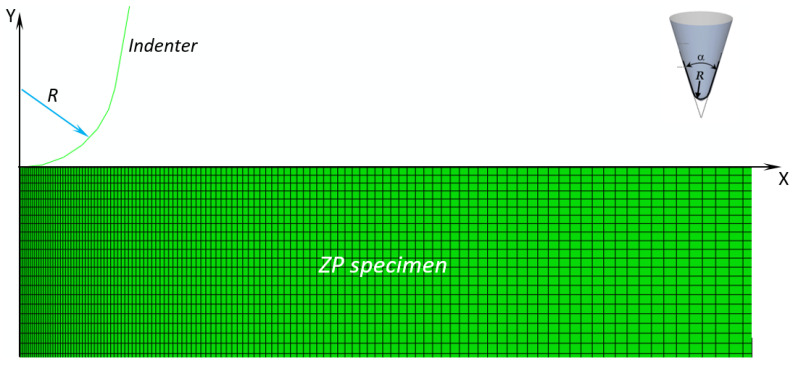
ABAQUS model simulating the nanoindentation process of the equine zona pellucida (ZP) specimens and 3D sketch of the blunt cone indenter geometry.

In this study, the nanoindentation experiments performed in [19] on the equine ZP specimens were simulated by a parametric axisymmetric finite element model using ABAQUS^®^ Version 6.12 (Dassault Systèmes, Vélizy-Villacoublay, France) software. The AFM tip was modeled as a rigid blunt cone while the ZP sample was modeled as an incompressible visco-hyperelastic slab. Following [14], the AFM tip radius of curvature *R* is 10 nm while the blunt cone angle of aperture *α* is set equal to 40° (see also the 3D sketch of the blunt cone indenter in the inset of Figure 1). The axis of symmetry Y of the FE model represents the indentation direction. By moving the rigid blunt cone in the negative Y-direction, the load is transferred to the ZP slab whose bottom edge is clamped and a reaction force is developed at the contact interface. 

Figure 1 shows the finite element model of the tested specimens: the mesh of the ZP membrane has about 70,000 four-node bilinear hybrid CAX4H elements (which allowed the incompressible behavior of the ZP material to be modeled) and 70,000 nodes. Mesh is properly refined in the contact zone between the indenter and the specimen. Convergence analysis was carried out in order to obtain mesh-independent solutions. 

Finite element analysis accounted for both geometric (i.e., large deformations) and material (i.e., hyperelasticity) nonlinearities. The automatic time-stepping option was chosen to speed up converge of nonlinear FE analysis. The contact interaction between AFM tip and soft ZP sample was hypothesized to be frictionless and the “hard contact” (i.e., no force is exchanged before surfaces come into contact) option available in ABAQUS was selected.

A total displacement *δ*_TOT_ = 300 nm was imposed on the rigid body part of the FE model corresponding to the AFM probe. The *δ*_TOT_ displacement was ramped over 1000 steps and ABAQUS computed the reaction force developed at the contact interface for each step, thus building the numerical force–indentation curve. As mentioned in Section 2.1, the actual indentation depth used in the AFM measurements ranged between 416 and 1020 nm for the different indentation rates. However, some nanoindentation curves recorded experimentally have a locally unstable behavior where the force–indentation trend is not strictly monotonic. To overcome this problem, indentation depth was limited to 300 nm in the FE simulations as all force–indentation curves remain strictly monotonic within this range. The selected 300 nm indentation range was indeed representative of the whole force–indentation curve obtained for each indentation rate. In fact, each *F*–*δ* curve recorded experimentally at a given indentation rate was fitted by the same power function *F* = *δ^β^* with an *R*^2^ of at least 0.95 regardless of having selected the 0 to 300 nm range or the whole indentation range.

The Young’s modulus of the silicon nitride AFM tip was set as 300 GPa. The equine ZP slab was modeled as a visco-hyperelastic material following the eight-chain Arruda–Boyce constitutive model [37]. This model relies on the statistical mechanics description of a material with a cubic representative volume element containing eight chains along diagonal directions. The strain energy function is expressed as:

(2) $$\begin{array}{ll} W=\mu_{8chain}\times & [\frac{1}{2}\left(\bar{I_{1}}-3\right)+\frac{2}{20\lambda_{L}^{2}}\left({\bar{I_{1}}}^{2}-9\right)+\frac{33}{1050\lambda_{L}^{4}}\left({\bar{I_{1}}}^{3}-27\right)+\frac{76}{7000\lambda_{L}^{6}}\left({\bar{I_{1}}}^{4}-81\right)\\ & +\frac{519}{673750\lambda_{L}^{8}}\left({\bar{I_{1}}}^{5}-243\right)] \end{array}$$

where *μ*_8*chain*_ is the shear modulus and *λ_L_* is the distensibility of the material; the first strain invariant is defined as $\bar{I_{1}}$ = tr[*C*] where [*C*] is the Cauchy–Green strain tensor. The Arruda–Boyce model is activated in ABAQUS by specifying the *μ*_8*chain*_ and *λ_L_* values as material parameters. The apparent Young’s modulus of the material is defined as *E*_AB_ = 2(1 + *ν*)*μ*_8*chain*_. The Poisson’s ratio is theoretically equal to 0.5 for incompressible materials but is set equal to 0.4999 in FE computations to guarantee numerical stability.

Other classical hyperelastic models, such as the two-parameter Mooney–Rivlin model, neo-Hookean model and Ogden’s constitutive law, were used to confirm that the Arruda–Boyce model actually is the best available option for modeling the nonlinear elastic behavior of the immature equine ZP. 

The Mooney–Rivlin model [38,39] is a very classical phenomenological model with the following strain energy function:

(3) $$W=C_{10}\left(\bar{I_{1}}-3\right)+C_{01}\left(\bar{I_{2}}-3\right)$$

where *C*_10_ and *C*_01_ are the MR constants given as input to ABAQUS as material properties. The second strain invariant is defined as $\bar{I_{2}}$ = {tr^2^[*C*] − tr^2^[*C*]^2^}. The apparent Young’s modulus of the material is defined as *E*_MR_ = 4(1 + *ν*)(*C*_10_+*C*_01_). The initial shear modulus is hence defined as *μ*_MR_ = 2(*C*_10_ + *C*_01_).

The neo-Hookean model [40,41] is based on statistical thermodynamics of cross-linked polymer chains. Since it can be derived from the Mooney–Rivlin model by setting *C*_01_ = 0, ABAQUS requires only one material parameter as input. The apparent Young’s modulus of the material is defined as *E*_NH_ = 4(1 + *ν*)*C*_10_. The initial shear modulus is hence defined as *μ*_NH_ = 2*C*_10_.

The 3rd-order Ogden’s hyperelastic law [42] implements the following strain energy density function depending on the principal stretches ${\bar{l}}_{1}$, ${\bar{l}}_{2}$ and ${\bar{l}}_{3}$ of the left Cauchy strain tensor:

(4) $$W=\sum_{i=1}^{3}\frac{\mu_{i}}{\alpha_{i}}\left({\bar{l}}_{1}{}^{\alpha_{i}}+{\bar{l}}_{2}{}^{\alpha_{i}}+{\bar{l}}_{3}{}^{\alpha_{i}}-3\right)$$

where the *μ*_1_, *α*_1_, *μ*_2_, *α*_2_, *μ*_3_ and *α*_3_ constants must be given as input to ABAQUS. The initial shear modulus of the material is defined as *μ*_OG_ = (*μ*_1_*α*_1_ + *μ*_2_*α*_2_ + *μ*_3_*α*_3_)/2.

The viscous behavior of the material was modeled in ABAQUS by using *N_Prony_*-term Prony series expansions of hyperelastic parameters. For the Mooney–Rivlin and neo-Hookean models, hyperelastic constants *C*_10_ and *C*_01_ are relaxed as (*i*,*j* = 0,1 with *i* ≠ *j*):

(5) $$C_{ij}^{R}\left(t\right)=C_{ij}\times \left[1-\sum_{j=1}^{N_{Prony}}g_{j}\times \left(1-e^{-\;\frac{t}{\tau_{j}}}\right)\right]$$

In the case of the Ogden model, hyperelastic constants *μ*_1_, *μ*_2_ and *μ*_3_ are relaxed as:

(6) $$\mu_{i}^{R}\left(t\right)=\mu_{i}\times \left[1-\sum_{j=1}^{N_{Prony}}g_{j}\times \left(1-e^{-\;\frac{t}{\tau_{j}}}\right)\right]\;\;(i\;=\;1,2,3)$$

Finally, for the Arruda–Boyce model, the effective relaxation modulus $\mu_{8chain}^{R}t$ is the product of the instantaneous shear modulus *μ_8chain_* by the dimensionless relaxation function:

(7) $$\mu_{8chain}^{R}\left(t\right)=\mu_{8chain}\times \left[1-\sum_{j=1}^{N_{Prony}}g_{j}\times \left(1-e^{-\;\frac{t}{\tau_{j}}}\right)\right]$$

In Equations (5)–(7), *g_j_* is the *j*-th Prony constant (*j* = 1, 2, …, *N_Prony_*) and *τ_j_* is the corresponding relaxation time constant.

In this study, the time-dependent visco-hyperelastic FE analyses were performed for each indentation rate *v_i_* (i.e., 0.5, 1, 2, 4, 6, 8 and 10 μm/s) by ramping the total displacement *δ*_TOT_ given to the AFM indenter over the *t_indent_i_*= *δ*_TOT_/*v_i_* time. 

### 2.3. Determination of Equine ZP Visco-Hyperelastic Properties

The visco-hyperelastic properties of the equine ZP specimens were determined by performing an inverse analysis based on nonlinear optimization. The difference between nanoindentation data gathered from AFM measurements and finite element results was minimized by perturbing the unknown material parameters included as optimization variables. In the optimization process, the computed force–indentation curve by ABAQUS for a given set of material properties was compared with the experimentally measured *F*–*δ* curve. The difference between numerical results and experimental data was expressed by the error functional Ω depending on unknown properties. Hence, when the ZP was hypothesized to follow the Arruda–Boyce hyperelastic model, the optimization problem took the following formulation:

(8) $$\left\{ \begin{array}{l} Min\left\{Ω\left(\mu_{8chain},\lambda_{L},G,T\right)=\sqrt{\frac{1}{N_{CNT}}\sum_{k=1}^{N_{CNT}}{\left(\frac{F_{FEM}^{k}-\bar{F^{k}}}{\bar{F^{k}}}\right)}^{2}}\;\right\}\\ 0.1\;kPa\leq \mu_{8chain}\leq 100\;kPa\;\;\;\;\\ 1\;\leq \lambda_{L}\leq 10\;\;\;\\ 10^{-7}\leq g_{1},g_{2},\ldots ,g_{NProny}\leq 1\;\\ 10^{-10}\leq \tau_{1},\tau_{2},\ldots ,\tau_{NProny}\leq 1\;\; \end{array}\right.$$

where: Ω is the error functional to be minimized, *μ*_8*chain*_ and *λ_L_* are shear modulus and the distensibility of the hyperelastic material, ***G***(*g*_1_, *g*_2,_ …, *g_NProny_*) and ***T***(*τ*_1_, *τ*_2_, …, *τ_NProny_*) are the Prony constants and the corresponding relaxation time constants for the viscous component. The selected bounds for *μ*_8*chain*_ cover the range of variation of the apparent Young’s modulus of equine oocytes indicated in [19] for the same range of indentation rates and account for the fact that elastic moduli determined under the assumption of hyperelastic behavior may be up to orders of magnitude smaller than those determined with the classical Hertzian model. Generally speaking, material property bounds set in inverse problems should always be large enough to maximize design freedom and quickly converge to the target solution.

In Equation (8), $F_{FEM}^{k}$ and $\bar{F^{k}}$, respectively, are the indentation force values for the k-th load step computed by ABAQUS and those measured experimentally by AFM. The number of control points *N_CNT_* corresponds to the number of load steps executed for completing the nonlinear FE analysis. 

A convergence limit *ε_CONV_*= 0.0001 (i.e., 0.01%) was set. For Ω < *ε_CONV_*, the identification process was automatically stopped at the current iteration and material properties were listed as output. Conversely, for Ω > *ε_CONV_*, the unknown material parameters *μ_8chain_*, *λ_L_*, (*g*_1_, *g*_2_, …, *g_NProny_*) and (*τ*_1_, *τ*_2_, …, *τ_NProny_*) were perturbed in the subsequent design cycles until reaching convergence.

Similar formulations of the inverse problem (8) were used for the other hyperelastic models considered in this study, but other variants of Equation (8) are not presented here in order to avoid repetition. The total number of design variables *NDV* of the inverse problem was (2 + 2 × *N_Prony_*) for the Mooney–Rivlin model, (1 + 2 × *N_Prony_*) for the neo-Hookean model, (6 + 2 × *N_Prony_*) for the Ogden model and (2 + 2 × *N_Prony_*) for the Arruda–Boyce model. Bounds of hyperelastic parameters set for each model allowed coverage of the same range of variation of Arruda–Boyce’s shear modulus and consequently the apparent Young’s modulus range of variation indicated in [19] for the equine ZP.

The inverse problem stated by Equation (8) was solved for each hyperelastic model and each indentation rate. The corresponding experimental force–indentation curve taken as a target in the optimization process was the average of the *F*–*δ* curves recorded at 50 different points of the ZP specimen. This strategy may be considered reliable because the standard deviation of measured AFM data with respect to the target average *F*–*δ* curve was always lower than 10% for all indentation rates. The target force–indentation curves were filtered to remove noise and make the identification process easier. The optimization runs were started from twenty sets of trial values of material properties randomly generated using the equation:

*x*_i_° = *x*_i_^L^ + *ρ*_i_ × (*x*_i_^U^ − *x*_i_^L^) (9)

where *x*_i_° is the initial value assigned to the i-th unknown material parameter, *ρ*_i_ is a random number in the (0,1) interval, *x*_i_^L^ and *x*_i_^U^ are the lower and upper bounds of the i-th unknown material parameter. 

The multi-start optimization strategy together with the large range of variability set for material parameters covered the whole search space, thus increasing the probability of finding the target solution. Serial optimization runs were carried out for each initial solution generated with Equation (9) to avoid premature convergence to false solutions: each run started from the optimal solution found in the previous run. For each initial solution, the search process ended when the relative variations of error functional ‖(Ω_K_ − Ω_K−1_)/Ω_K−1_‖ and design vector ‖***X***_K_ − ***X***_K−1_‖/‖***X***_K−1_‖ between the last two serial runs became smaller than 10^−^^7^.

The optimization problem stated in Equation (8) was solved with the sequential quadratic programming (SQP) method, a globally convergent gradient-based optimization algorithm, available in MATLAB^®^ 7.0 commercial software (MathWorks Inc., Austin, TX, USA). SQP [43,44] iteratively solves a series of approximate sub-problems, each of which formed by a quadratic approximation of the cost function and linear approximations of the optimization constraints. The cost function (in the present case, the error functional Ω of Equation (8)) is minimized by perturbing the design point ***X*_k_** of the current iteration along the search direction ***S***. The approximate sub-problem is hence defined as:

(10) $$\underset{S}{\mathrm{Min}}\;\left\{Ω\left(X_{k}\right)+S^{T}\bar{\nabla }Ω\left(X_{k}\right)+\frac{1}{2}S^{T}\left[B\right]S\right\}$$

where the search direction ***S*** is obtained by solving the approximate sub-problem formulated in the current iteration. The [*B*] matrix is initially set equal to the identity matrix of dimension *NDV* and updated in each iteration until it coincides with the Hessian matrix of Lagrangian function ℒ (***X***,***Λ***) of the error functional Ω when the SQP algorithm converges to the optimal solution (i.e., the values of material properties to be identified). The Lagrangian function ℒ (***X***,***Λ***) is defined as:

(11) $$L\;(X,\Lambda )\;=\;\left\{Ω\left(X\right)+\sum_{i=1}^{NC_{+}}\lambda_{i}{}^{+}\times UB_{i}-\sum_{i=1}^{NC_{-}}\lambda_{i}{}^{-}\times LB_{i}\right\}$$

where the ***X*** vector includes the *NDV* material parameters to be identified while the ***Λ*** vector includes *NDV/2* Lagrange multipliers $\lambda_{i}{}^{+}\;$ and *NDV/2* Lagrange multipliers $\lambda_{i}{}^{-}$ corresponding, respectively, to upper and lower bounds of design variables. The *NC*_+_ = *NC*_−_ = *NDV/2* equality holds true because Equation (8) includes one ≤0 type constraint (i.e., *x*_i_ ≤ *x*_i_^U^ for the upper bound, which is rearranged as *UB*_i_ = *x*_i_ − *x*_i_^U^ ≤ 0) and one ≥0 type constraint (i.e., *x*_i_ ≥ *x*_i_^L^ for the lower bound, which is rearranged as *LB*_i_ = *x*_i_ − *x*_i_^L^ ≥ 0) for each material parameter to be identified.

The identification algorithm was coded in the MATLAB^®^ software environment and entailed the following steps for each indentation rate considered in this study: (i) define design variables (i.e., unknown material properties) and their corresponding lower and upper bounds; (ii) generate a random initial design; (iii) use the “*fmincon*” command to perform the SQP optimization search in MATLAB; (iv) use the ABAQUS solver to perform the linear finite element analyses entailed by the SQP process; (v) check for convergence of the SQP optimization process; (vi) check for convergence of multi-start optimization search; (vi) output identified material properties.

In the present study, all optimization runs performed by MATLAB were completed within about 20 iterations. The computational cost of MATLAB optimizations (given by the number of evaluations of the error functional Ω) never exceeded 30 × (*NDV* + 1) evaluations, where (*NDV* + 1) evaluations served for computing the gradient of cost functional with respect to unknown material parameters in each iteration while the remaining evaluations were performed to minimize the approximate cost function on the search direction ***S*** obtained from the approximate sub-problem.

### 2.4. Sensitivity Analysis on the Relationship between Probe Geometry and Indentation Rate

In order to study the sensitivity of an immature equine ZP’s indentation response to AFM probe geometry, the average material properties extracted from the inverse analysis carried out at different indentation rates were given as input to three ABAQUS models where the radius of curvature *R* of the AFM tip was 10 nm, 30 nm and 50 nm. The total angle of aperture of the probe was fixed at 40° in all finite element simulations, consistent with the nominal tip geometry adopted in the nanoindentation measurements of [19]. The 30 and 50 nm radii were considered here because it may happen that the AFM tip flattens if the force–indentation curve selected as a target for the inverse analysis results from the average of many *F*–*δ* curves recorded over a selected area of the specimen surface. The effect of variations in AFM tip geometry in the course of nanoindentation measurements has not often been addressed in the literature.

Furthermore, in order to investigate the relationship between probe shape and indentation rate, for each AFM tip radius, the rate was set equal to 0.1 μm/s, 1 μm/s, 10 μm/s and 100 μm/s, thus covering a 50 times broader range than the one considered in the nanoindentation experiments (i.e., 0.5–10 μm/s). Following the steps explained in [13], for each tip radius, it was attempted to determine the limit indentation rate *v*_i,lim_ below which it is possible to neglect viscous terms and carry out the characterization process of the immature equine ZP by considering only the hyperelastic component. For that purpose, the average material parameters extracted from inverse analysis were given as input to the visco-hyperelastic and the purely hyperelastic model. The force–indentation curve *F*–*δ* was computed by ABAQUS for each indentation rate and the error $\varepsilon_{F_{max}}$ on the maximum indentation force developed for *δ*_TOT_ = 300 nm was defined as:

(12) $$\varepsilon_{F_{max}}=\left|\frac{F_{\delta_{TOT}}^{visco-hyperelastic}-F_{\delta_{TOT}}^{hyperelastic}}{F_{\delta_{TOT}}^{hyperelastic}}\right|\;\times \;100$$

The forces $F_{\delta_{TOT}}^{visco-hyperelastic}$ and $F_{\delta_{TOT}}^{hyperelastic}$ were computed by ABAQUS by integrating contact stresses developed at the probe/specimen interface, respectively, for the visco-hyperelastic and the purely hyperelastic models. Maximum indentation force obviously occurs at *δ*_TOT_. The $\varepsilon_{F_{max}}$(*v*_i_) function was then fitted by a 3rd-order polynomial passing through the four values $\varepsilon_{F_{max}}$(0.1), $\varepsilon_{F_{max}}$(0.5), $\varepsilon_{F_{max}}$(10) and $\varepsilon_{F_{max}}$(100) computed for each indentation rate. Similar to [13], at the limit indentation rate, the error $\varepsilon_{F_{max}}\;$ on maximum indentation force must be at most 25%. Hence, in this study, the limit indentation rate was obtained by solving the equation $\varepsilon_{F_{max}}$(*v*_i,lim_) = 25 in the [0.1,100] μm/s range.

## 3. Results and Discussion

The first issue considered in this study was to check if the Arruda–Boyce model is actually the best hyperelastic model for the immature equine ZP. For that purpose, the *F*–*δ* curve relative to the lowest indentation rate of 0.5 μm/s was considered and the corresponding inverse problem was solved for the Arruda–Boyce, two parameter Mooney–Rivlin, neo-Hookean and Ogden constitutive models. Interestingly, none of these models performed well when only one Prony term was included in the analysis. Arruda–Boyce was the best model, followed by Ogden, Mooney–Rivlin and neo-Hookean. However, the *R*^2^ correlation coefficient achieved using the Arruda–Boyce model was only 0.6495, while the other models did not go beyond *R*^2^ = 0.55. Interestingly, increasing the number of unknown material parameters, as happens for the Ogden model, should have increased design freedom and in principle have allowed a better fitting of AFM data. However, it actually complicated the search process, making the SQP optimizer remain stuck at local minima. The same ranking of constitutive models was observed for the very high indentation rate of 10 μm/s and the Arruda–Boyce model was confirmed to be the best hyperelastic model overall; again, the other models achieved lower values of *R*^2^ than the Arruda–Boyce model, always below 0.475. As expected, the quality of fitting became worse as the viscous effects were more pronounced due the higher indentation rate but still only one Prony term was included in the analysis. Ranking of constitutive models for intermediate indentation rates obviously coincided with those seen for the maximum and minimum rates.

Once the Arruda–Boyce model was proven to be the best hyperelastic model, attention was focused on the accurate description of the viscous response of the immature equine ZP. Table 1 presents the values of material parameters obtained by solving the inverse problem of Equation (8) for the different indentation rates including one or two terms in the Prony series.

It can be seen that the two-term Prony series described the visco-hyperelastic response of the equine ZP with a good level of accuracy. In fact, the correlation coefficient ranged between 0.9307 and 0.9506, randomly fluctuating about the average value of 0.9412. Conversely, in the case of single-term Prony series, the *R*^2^ coefficient exhibited a decreasing trend for the higher indentation rates. Such a behavior is very logical if one considers that the single-term Prony series could not efficiently capture viscous effects and hence its efficiency must decrease as the analysis passes from “quasi-static” conditions (i.e., low indentation rates) to “fully dynamic” conditions (i.e., high indentation rates). 

To confirm the inherent validity of the Arruda–Boyce constitutive model in describing the mechanical response of the equine ZP, the identification process was also performed using the Ogden model. Interestingly, values identified for the *μ*_i_ and *α*_i_ parameters of the Ogden model led to determining values of shear modulus *μ*_OG_ = (*μ*_1_*α*_1_ + *μ*_2_*α*_2_ + *μ*_3_*α*_3_)/2 at most 15% different from the *μ*_8*chain*_ shear modulus identified by the Arruda–Boyce model. However, relaxation times were much more dispersed than for the Arruda–Boyce model and the highest correlation achieved by the Ogden model was *R*^2^ = 0.725. In summary, the visco-hyperelastic behavior of the equine ZP was inherently described by the Arruda–Boyce model and the fitting accuracy did not depend just on a favorable combination of mathematical conditions. The present study hence fully satisfies the most important rule in the identification of nonlinear materials: correlation between outputs of numerical models and experimental data must be based on the effective ability of the constitutive model to capture the mechanical behavior of the investigated material.

Table 1 shows that the identification process implemented in this research was very robust. The average values of shear modulus and distensibility obtained for the two-term Prony model are 1.2507 kPa and 1.2790, respectively. The standard deviations on *µ*_8*chain*_ and *λ_L_* are, respectively, 0.01578 kPa and 0.01893 and, hence, less than 1.5% of the corresponding average values. Conversely, the single-term Prony model was much less robust, showing a dispersion of about 5.8% on the shear modulus value. The dispersion on the first relaxation time *τ*_1_ was raised to about 20% in the case of the single-term Prony model vs. only 5.6% of the two-term Prony model. A fairly large dispersion was also observed for the second relaxation time *τ*_2_ of the two-term model, 13.9%, yet this was smaller than its counterpart for the *τ*_1_ constant of the single-term model.

**Figure 2:**
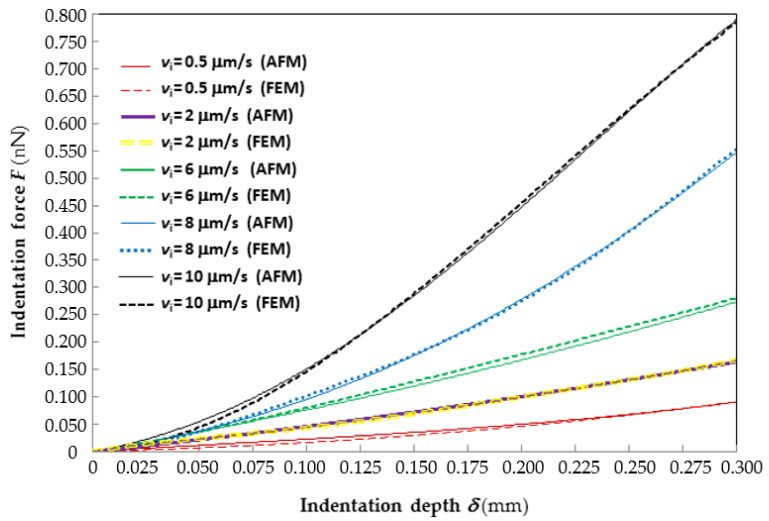
Comparison of representative experimental force–indentation curves with those reconstructed via inverse analysis for different indentation rates.

Figure 2 shows the good agreement between the average force–indentation curves recorded experimentally at different indentation rates and their counterparts reconstructed by ABAQUS via inverse analysis. For clarity of representation, only the *F*–*δ* curves corresponding to the lowest (0.5 μm/s), 2nd lowest (2 μm/s), median (6 μm/s), 2nd largest (8 μm/s) and largest (10 μm/s) indentation rates are plotted in the figure. The *F*–*δ* curves relative to the 4 μm/s rate almost coincide with those relative to the 6 μm/s rate and hence are not shown. Interestingly, the “2 μm/s” *F*–*δ* curves practically average those relative to the lowest and median indentation rates; the “8 μm/s” curve practically averages those relative to the median and highest indentation rates. The horizontal axis of the plot is limited to the indentation range of 300 nm considered in the identification process.

Since the data plotted in the figure are very dense, error bars are not shown. However, it can be seen from the figure that the experimental *F*–*δ* curve relative to the lowest indentation rate (i.e., 0.5 μm/s) stands above the numerical curve over the whole indentation range; the largest force gap is about 22% at *δ* = 0.11 μm. The *F*–*δ* curves relative to the 2nd lowest indentation rate (i.e., 2 μm/s) practically overlap and the force gap never exceeds 1%. The experimental *F*–*δ* curve relative to the median indentation rate (6 μm/s) always stands below the numerical curve; the largest force gap is only 2.55% at *δ* = 0.3 μm. The *F*–*δ* curves relative to the highest indentation rate (i.e., 10 μm/s) cross each other at two points (i.e., *δ* = 0.125 and 0.265 μm); the largest force gap is 18% at *δ* = 0.045 μm. The *F*–*δ* curves relative to the 2nd highest indentation rate (i.e., 8 μm/s) cross each other at three points (i.e., at *δ* = 0.05, 0.163 and 0.245 μm); the largest force gap is 6.6% at *δ* = 0.095 μm. It should be noted that error peaks are “localized” in a very small fraction of the indentation range and hence they marginally affect the overall accuracy of the fitting stated by the *R*^2^ coefficient.

The results of the characterization process appear to be consistent with the experimental observations reported in [19]. The equine ZP is softer than the porcine ZP but the difference in stiffness between the two materials is much smaller than the one order of magnitude found in [19]. In fact, the apparent Young’s modulus of the Arruda–Boyce model computed as *E*_AB_ = 2(1 + *ν*)*μ*_8*chain*_ by using the average value *μ*_8*chain*_ = 1.251 kPa from the results of Table 1 is about 3.75 kPa for the equine ZP vs. 5.05 kPa obtained for the porcine ZP considering the 1.688 kPa average shear modulus reported in [29]. Such a drastic reduction of the stiffness difference between the equine and porcine ZPs is related to the significantly lower distensibility of the polymeric network of the equine ZP with respect to the porcine ZP: on average, only 1.279 vs. 2.958 indicated in [29]. Hence, the elastic component of the mechanical response of the equine ZP tends to be much more nonlinear than its counterpart for the porcine ZP. Viscous effects also are more pronounced in the case of equine ZP as a two-term Prony series was necessary, while a single-term Prony series was enough for the porcine ZP [13,29].

In summary, the two ZPs exhibited a significantly dissimilar mechanical behavior and such a difference could be captured only by modeling time dependency in an accurate way. Conversely, the simplified viscoelastic model considered in [19] highlighted a linear dependency of elastic modulus with respect to indentation rate for both ZPs and stiffness values of the two ZPs identified at each indentation rate were always in the same ratio, thus leading us to conclude that, practically, the two materials have a very similar mechanical behavior.

The differences between the visco-hyperelastic response of immature equine and porcine ZPs highlighted above may be explained in view of the studies on the morphological structures of these cellular membranes carried out by Mugnier et al. [45]. In fact, electron microscopy investigations revealed that the equine ZP has a more compact structure with much smaller pores than the porcine ZP. Hence, the polymeric network of the equine ZP has short chains (i.e., with close cross-link points), which can be extended much less than those of the porcine ZP. This also results in a more significant viscous behavior of the equine ZP: the shorter distance between polymeric chains makes it easier to trigger local chain slipping phenomena once an external perturbation is transferred to the ZP structure. This statement is also supported by the lower value of shear modulus identified for the equine ZP with respect to the porcine ZP: only 1.251 kPa vs. 1.688 kPa. Hence, slippage of polymeric chains is more likely to occur in the case of equine ZP because of the lower shear stiffness of this material. In summary, the equine ZP membrane has a structure formed by shorter (this is proven by the shorter inter-pore distance observed for the equine ZP) and softer (this is proven by the smaller values of elastic moduli identified for the equine ZP) polymeric chains than the porcine ZP membrane. Since distensibility of equine ZP is small, the viscous component must drive the overall deformation.

**Figure 3:**
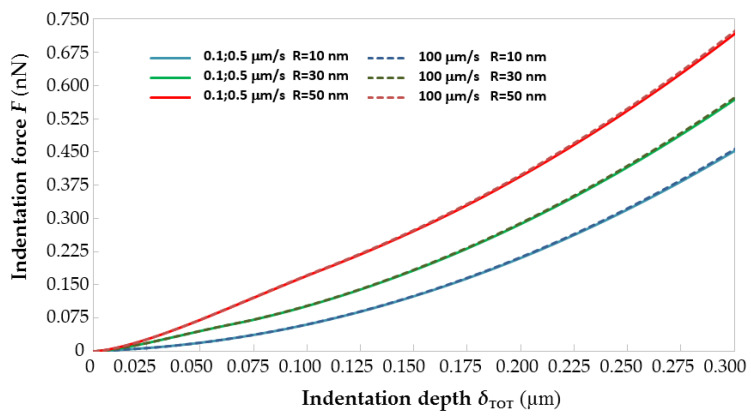
Effects of AFM probe geometry and indentation rate on the visco-hyperelastic response of the equine ZP.

The sensitivity analysis carried out to assess the relationships between indentation rate and AFM probe geometry confirms the arguments developed above. Figure 3 shows the force–indentation curves generated by ABAQUS by giving as input to the FE models built for *R* = 10, 30 and 50 nm and *v*_i_ = 0.1, 0.5, 10 and 100 μm/s the average visco-hyperelastic properties derived from Table 1 for the two-term Prony model. For each value of tip radius *R*, the curves plotted in Figure 3 for the 0.1 and 0.5 μm/s rates are averaged to make the figure more readable. It can be seen that *F*–*δ* curves shift up as the AFM tip radius increases: basically, indentation forces grow for larger probes because of the reduction of contact pressure caused by the increase in contact area. Such a behavior is fully consistent with the results of [13,16] and is well documented in any textbook of contact mechanics.

Figure 3 shows that the *F*–*δ* curves of immature equine ZP evaluated for the average material properties are insensitive to the indentation rate *v*_i_. In fact, the largest difference in the maximum indentation force (evaluated at the indentation depth of 0.3 μm) when *v*_i_ passes from 0.1 to 100 μm/s is 0.658%, 0.524% and 1.11%, respectively, for *R* = 10 nm, *R* = 30 nm and *R* = 50 nm. Regardless of having varied the AFM tip radius of curvature from 10 to 30 or 50 nm, it was not possible to find any limit indentation rate, even when reducing the indentation depth to 100 or 200 nm from the 300 nm range originally considered in the identification process. This confirms that the mechanical response of the equine ZP is characterized by a very significant viscous component. However, the lack of a limit indentation rate range for the equine ZP was somehow also expected considering that the corresponding range for a less viscous material like the porcine ZP varied from 180 to 430 nm/s and, hence, very close to static indentation conditions. This also explains the linear relationship between *v*_i,lim_ and the radius of curvature highlighted in [30].

## 4. Conclusions

This paper presented a very detailed study on the visco-hyperelastic characterization of the immature equine ZP. The main challenge was to assess the highly nonlinear behavior of the tested material. The Arruda–Boyce model was combined with two-term Prony series in order to simulate the nanoindentation response of ZP specimens tested at different indentation rates. Material properties were identified via an inverse analysis approach based on nonlinear optimization. The results were fully consistent with experimental evidence and the proposed approach proved itself very robust. Remarkably, the force–indentation curves recorded from AFM measurements were fitted by the inverse analysis, reaching an average value of correlation coefficient *R*^2^ = 0.9412. 

The identified mechanical behavior for equine ZP was critically compared with that of porcine ZP. An important conclusion achieved by this study is that the two materials have a very dissimilar visco-elastic response. This was found by properly modeling time-dependent behavior while a simplified visco-elastic model led us to conclude that the stiffness ratio for the two ZPs is independent of indentation rate. Although in this study two known materials (i.e., the immature equine and porcine ZPs) were analyzed with well-established methods (i.e., optimization-based inverse analysis taking AFM nanoindentation measurements as target data), the results discussed in the article should be considered novel and very significant for at least two reasons: (i) the mechanical response of two important biological soft materials such as the equine and porcine ZPs is now assessed at a high level of detail; (ii) it is illustrated how to compare the mechanical behavior of soft materials in the visco-hyperelastic regime. 

Interestingly, the experimental data gathered from nanoindentation experiments were fitted by a continuum mechanics model and the mechanical properties thus obtained were also interpreted by considering the real structure of the material detected by electron microscopy. Such a multi-scale approach should always be utilized in the mechanical characterization of highly nonlinear materials with an inherently complex structure, such as cells. The investigation phases illustrated in the article are absolutely general and represent the framework to be followed in the mechanical characterization of any soft polymeric material used in technological applications.

Future research should be directed at developing more effective nonlinear visco-hyperelastic models that also account for the real structure of the material at hand. In this regard, the continuum mechanics model adopted here for the equine ZP should be replaced by a discrete mechanistic model accounting for the interactions between cell parts that contribute to the viscous response and their counterpart driving the nonlinear elastic response. Speaking more generally, this approach is very useful to identify the mechanical behavior of any composite material comprising different phases that mutually interact.

## Figures and Tables

**Table 1:** Results of immature equine ZP identification process for the Arruda–Boyce model.

*v*_i_ (μm/s)	*N_Prony_*	*µ*_8*chain*_ (kPa)	*λ_L_*	*g*_1_	*τ*_1_ (s)	*g*_2_	*τ*_2_ (s)	*R* ^2^
0.5	1	1.3246	1.4656	0.4910	0.002549			0.6495
	2	1.2313	1.2613	0.4806	0.001160	0.4790	0.001162	0.9403
2	1	1.4048	1.4746	0.5958	0.002404			0.6579
	2	1.2598	1.2530	0.4939	0.001057	0.4911	0.001366	0.9307
4	1	1.3656	1.4010	0.5667	0.003189			0.5651
	2	1.2379	1.2766	0.4924	0.001002	0.4906	0.001000	0.9361
6	1	1.2037	1.4265	0.4966	0.002807			0.5194
	2	1.2532	1.2865	0.4940	0.001001	0.5010	0.001000	0.9489
8	1	1.3168	1.4243	0.5153	0.003586			0.6131
	2	1.2752	1.2962	0.5094	0.001073	0.4821	0.001287	0.9408
10	1	1.2377	1.4101	0.4966	0.003945			0.5583
	2	1.2468	1.3001	0.5133	0.001026	0.4808	0.001344	0.9506

## Data Availability

Data on force-indentation curves available from [19]. Data on numerical analysis and optimization available upon request.
